# Gene Expression Reprogramming by Citrate Supplementation Reduces HepG2 Cell Migration and Invasion

**DOI:** 10.3390/ijms25126509

**Published:** 2024-06-13

**Authors:** Rocchina Miglionico, Ilenia Matera, Giovanna Maria Ventola, Giovanna Marchese, Vittorio Abruzzese, Magnus Monné, Angela Ostuni, Faustino Bisaccia

**Affiliations:** 1 Department of Sciences, University of Basilicata, 85100 Potenza, Italy; rocchina.miglionico@virgilio.it (R.M.); ilenia.matera@unibas.it (I.M.); v.abruzz@hotmail.it (V.A.); magnus.monne@unibas.it (M.M.); 2 Genomix4Life Srl, 84081 Baronissi, Italy; giovanna.ventola@genomix4life.com (G.M.V.); giovanna.marchese@genomix4life.com (G.M.); 3 Genome Research Center for Health-CRGS, 84081 Baronissi, Italy

**Keywords:** HepG2 cells, citrate, RNA-seq, GSEA, tumor aggressiveness, cytochrome P450 family

## Abstract

Citrate, which is obtained from oxaloacetate and acetyl-CoA by citrate synthase in mitochondria, plays a key role in both normal and cancer cell metabolism. In this work, we investigated the effect of 10 mM extracellular citrate supplementation on HepG2 cells. Gene expression reprogramming was evaluated by whole transcriptome analysis using gene set enrichment analysis (GSEA). The transcriptomic data were validated through analyzing changes in the mRNA levels of selected genes by qRT-PCR. Citrate-treated cells exhibited the statistically significant dysregulation of 3551 genes; 851 genes were upregulated and 822 genes were downregulated. GSEA identified 40 pathways affected by differentially expressed mRNAs. The most affected biological processes were related to lipid and RNA metabolism. Several genes of the cytochrome P450 family were upregulated in treated cells compared to controls, including the *CYP3A5* gene, a tumor suppressor in hepatocellular carcinoma (HCC) that plays an important protective role in HCC metastasis. The citrate-induced dysregulation of cytochromes could both improve the effectiveness of chemotherapeutics used in combination and reduce the aggressiveness of tumors by diminishing cell migration and invasion.

## 1. Introduction

Over the past decade, many advances have been made to fight cancer. Biological drugs and drug delivery technologies are currently available and have improved patient survival, but, unfortunately, cancer continues to be a leading cause of death worldwide, accounting for nearly 10 million deaths in 2020. Liver cancer was the sixth most common malignancy and the third leading cause of cancer-related death in 2020 worldwide [1]. Hepatocellular carcinoma (HCC) is a primary liver cancer that predominantly arises in the context of chronic liver disease and cirrhosis, mostly caused by viral hepatitis, metabolic syndrome or alcohol abuse [2,3]. The etiology of HCC varies significantly by geography, age, ethnicity and the associated disease states [4]. HCC diagnosis typically involves a combination of imaging, biopsy and serologic tests [5]. HCC is also associated with extrahepatic metastases in the lungs, regional lymph nodes, bone and peritoneum/omentum, which result in poor prognoses [6]. Because HCC is usually diagnosed at an advanced stage, its management is complex and effective treatment options remain limited [7]. Molecular-targeted therapy with sorafenib, a multi-target tyrosine kinase inhibitor, is the first-line treatment approved for advanced HCC, either in a single or combinatory regimen with monoclonal antibodies. However, the development of drug resistance within 6 months remains a serious obstacle that must be overcome for HCC therapy. For years, several approaches have been employed to sensitize resistant cancer cells by chemotherapy, but with limited success; therefore, it is necessary to develop new and effective therapeutic targets and combination strategies [8,9]. In this regard, numerous studies have underlined the importance of providing new insights into the molecular regulation of reactive oxygen species (ROS) in order to develop novel treatment strategies to reactivate programmed cell death or overcome drug resistance, including in HCC cells [10,11].

Metabolic reprogramming and epigenetic modifications are now recognized as hallmarks of cancer because they allow cancer cells to sustain uncontrolled proliferation during tumor progression. Epigenetic regulation, including DNA methylation, histone modification and chromatin remodeling, contribute to metabolic reprogramming, modifying the expression levels of metabolic enzymes, which play a central role in glucose and amino acid metabolism and in lipid synthesis [12,13,14]. For example, under sufficient oxygen conditions, cancer cells display enhanced glycolysis and the increased secretion of lactate, instead of oxidative phosphorylation (Warburg effect or aerobic glycolysis) [15,16]. Furthermore, several studies have shown that the epigenome is sensitive to metabolic changes and that some metabolic intermediates provide substrates or cofactors for epigenetic modifications, which influence different molecular processes, such as gene transcription, DNA damage repair and replication [17,18]. Therefore, reprogramming the metabolism and modulating epigenetic mechanisms may be useful in fighting tumors, including HCC [13,19].

Citrate, which is an important intermediate in the tricarboxylic acid (TCA) cycle that arises from the metabolism of glucose and, in a lesser amount, from glutamine, links the cellular metabolism to histone acetylation [20]. Citrate is released into the cytoplasm through the mitochondrial citrate transporter SLC25A1, a member of the solute carrier transporter family that operates as a citrate/malate exchanger [21,22]. Moreover, exogenous citrate can be transported across the plasma membrane through the sodium-dependent transporter SLC13A5 [23]. When the ATP levels are high, citrate exerts negative feedback regulation on glycolysis and the TCA cycle itself. In addition, within the cytosol, both exogenous and endogenous citrate are cleaved by ATP citrate lyase (ACLY) into oxaloacetate (OAA) and acetyl-CoA. The latter is a precursor for the biosynthesis of fatty acids and it is also used for histone acetylation by histone acetyltransferases (HATs), which regulate gene expression by adding acetyl groups to histone residues [24].

Although numerous studies have been carried out on the effect of citrate on tumors, both in vivo and in vitro, the results obtained have not always given rise to the same conclusions and depend on the type of tumor and the concentration of exogenous citrate used [20,25,26]. However, there is growing evidence suggesting that the antitumor effect of exogenous citrate at high concentrations may be due to the regulation of some key enzymes of glycolysis, the TCA cycle, gluconeogenesis and fatty acid synthesis [27,28]. Preclinical experiments performed with in vitro and in vivo models demonstrated that high concentrations of citrate have an antitumor effect through various mechanisms, such as the inactivation of PFK1 and PFK2 [29], the inhibition of glycolysis and ATP production and the stimulation of apoptotic cell death by various processes [30,31,32,33]. Moreover, citrate inhibits the insulin-like growth factor-1 receptor (IGF-1R) pathway and activates PTEN, overall inhibiting the PI3K/Akt pathway, and increases the sensitivity of cells to cisplatin and to Bcl-XL inhibitors (ABT 737 or siXL1) [27]. Considering the crucial role of citrate in supplying the acetyl-CoA pool for fatty acid synthesis, as well as histone acetylation, in tumors [34,35,36], it is reasonable to believe that citrate treatment could promote metabolic reprogramming and epigenetic alterations able to modify the cellular phenotype and, in turn, cells’ sensitivity to anticancer drugs. Although citrate treatment has been shown to have many effects on different levels in tumor cells, the exact or major mechanism is still poorly understood.

Lastly, it is also necessary to consider that citrate not only has strong inhibitory effects on tumor growth and tumor cell proliferation, but it can also modulate macrophages, dendritic cells and T cells in the tumor microenvironment, suggesting that reprogramming the metabolism of these cells could provide a further effective therapeutic strategy [37].

In order to understand the effects of citrate better, in the present study, we assessed the effect of exogenous citrate supplementation on the transcriptome of the human hepatoma cell line HepG2. The results obtained demonstrated the extensive remodeling of the gene expression associated with several pathways; among them, many members of the cytochrome P450 family were upregulated. This novel finding suggests new molecular targets for potential anticancer drugs, and the results confirm the anticancer activity of citrate at a concentration of 10 mM as it slows down cell migration and invasion.

## 2. Results

### 2.1. Whole Transcriptome Analysis of the Citrate-Treated HepG2 Cell Line

Citrate plays roles in various biological processes, including cancer metabolism and regulation. Consistent with the role of citrate in regulating significant metabolic pathways and supplying acetyl groups for lipid synthesis and histone acetylation, including in cancer cells, it seemed appropriate to assess the whole transcriptome of hepatocarcinoma cells treated with citrate.

**Figure 1:**
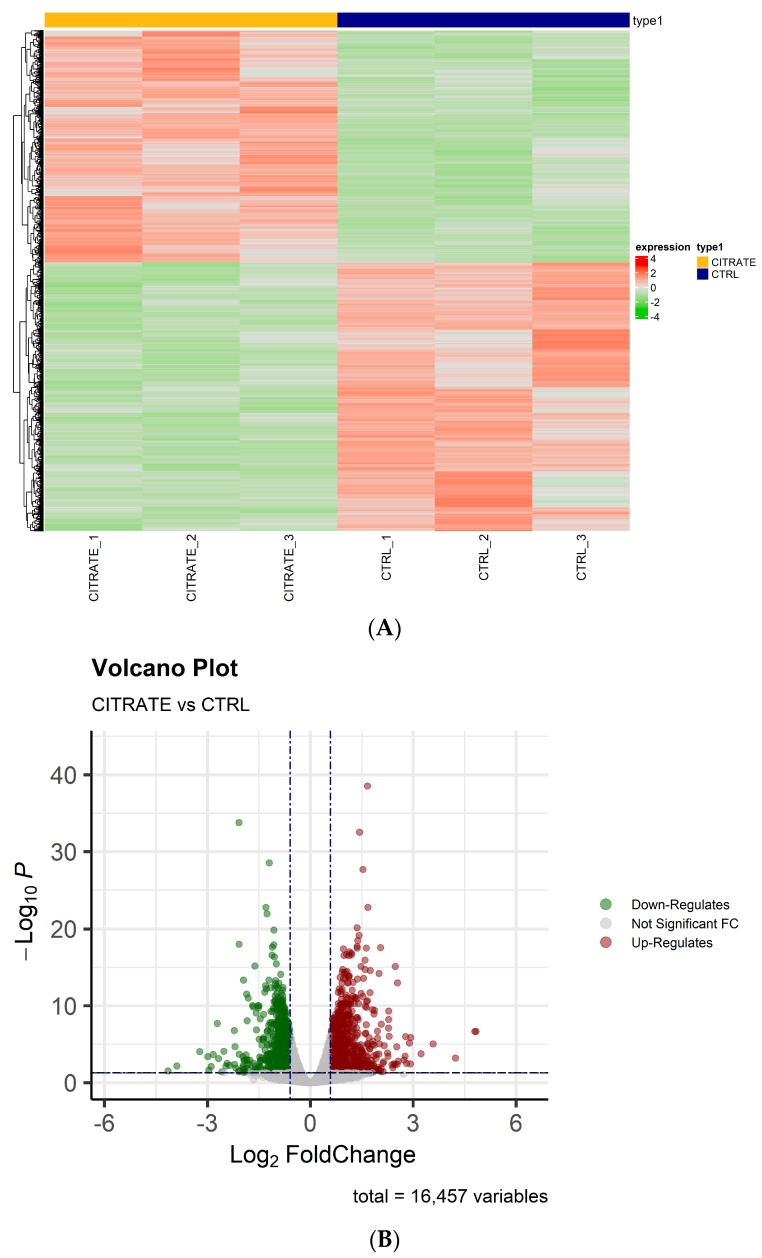
The analysis of differentially expressed genes. (**A**) A heatmap showing the relative expression of 3551 RNAs with padj ≤ 0.05 in citrate-treated cells compared to controls. The clustering was assessed using the “Euclidian” distance and the dendrogram clustering was obtained. The upregulated genes are shown in red, while downregulated genes are shown in green. (**B**) A volcano plot of differentially expressed genes (padj ≤ 0.05 and |FC| ≥ or ≤|1.5|).

Whole transcriptome RNA sequencing (RNA-seq) was performed by next-generation sequencing in HepG2 cells treated with 10 mM citrate and untreated cells to highlight possible gene differences between the two conditions. After filtering out poor-quality reads and trimming the adaptors, the obtained reads were aligned against the human genome reference (HG38—Release 37 (GRCh38.p13)). Principal component analysis (PCA) revealed good variance between the two condition groups and showed complete separation between the samples’ conditions. On the basis of the obtained gene counts, in total, we detected 16,457 expressed genes in all samples. To investigate the overall gene expression differences between the treated and control groups, hierarchical clustering analyses were performed. The heatmap showed that the trend in the gene expression change was consistent in the two groups, and the differences between the two conditions were statistically significant (Figure 1A). The differential expression analysis revealed a high number of dysregulated genes that were statistically significant (padj ≤ 0.05)—precisely 3551—between the two groups (citrate-treated vs. control). Among them, 851 genes were significantly (padj ≤ 0.05 and |FC| ≥ 1.5) upregulated and 822 genes (padj ≤ 0.05 and |FC| ≤ −1.5) were significantly downregulated (Table S1).

As shown in the volcano plots, the differentially regulated genes in the treated cells differed significantly from those found in the controls (Figure 1B). The raw data and the normalized counts of genes identified are available in the ArrayExpress repository under accession number E-MTAB-13852.

### 2.2. Gene Set Enrichment Analysis (GSEA)

In order to investigate the pathways affected by differentially expressed mRNAs (DEmRNAs) and assess which pathways might be significantly modulated in HepG2 cells treated for 24 h with citrate 10 mM, we performed gene set enrichment analysis (GSEA), a computational enrichment method that evaluates RNA-seq data at the level of gene sets. In fact, GSEA determines whether a defined gene set can show statistically significant, concordant differences between two biological states. The gene sets on the GSEA-MSigDB file servers were from the Molecular Signature Database (MSigDB) and the list of genes with HGNC gene symbols. In the MSigDB, we selected the Gene Ontology C5 module to query all ontologies: molecular function (MF), biological process (BP) and cellular component (CC).

Our dataset had 3551 native features. After collapsing the features into gene symbols, there were 3453 genes identified that were used in the analysis. By default, GSEA ignores gene sets that contain fewer than 15 genes or more than 500 genes (min = 15, max = 500). The analysis report contained two “Enrichment in Phenotype” sections. The first section showed results for gene sets that had a positive enrichment score (gene sets that showed enrichment at the top of the ranking list) and the second section showed results for gene sets that had a negative enrichment score (gene sets that showed enrichment at the bottom of the ranking list).

In detail, 1732/3453 gene sets were upregulated in the phenotype “na_neg” or gene sets that had a negative enrichment score (gene sets that showed enrichment at the bottom of the ranking list). Of these gene sets, 721 gene sets were significantly enriched at an FDR < 25%, 365 at a nominal *p*-value < 1% and 573 at a nominal *p*-value < 5% (Table S2). Similarly, 1721/3453 gene sets were upregulated in the phenotype “na_pos” or gene sets that had a positive enrichment score (gene sets that showed enrichment at the top of the ranking list). We found that 50 gene sets were significantly enriched at an FDR < 25%, 66 at a nominal *p*-value < 1% and 220 at a nominal *p*-value < 5% (Table S3).

**Figure 2:**
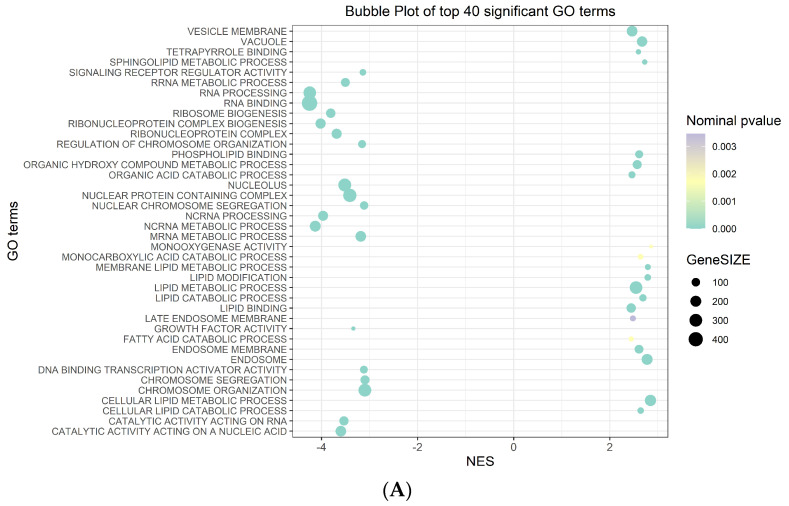

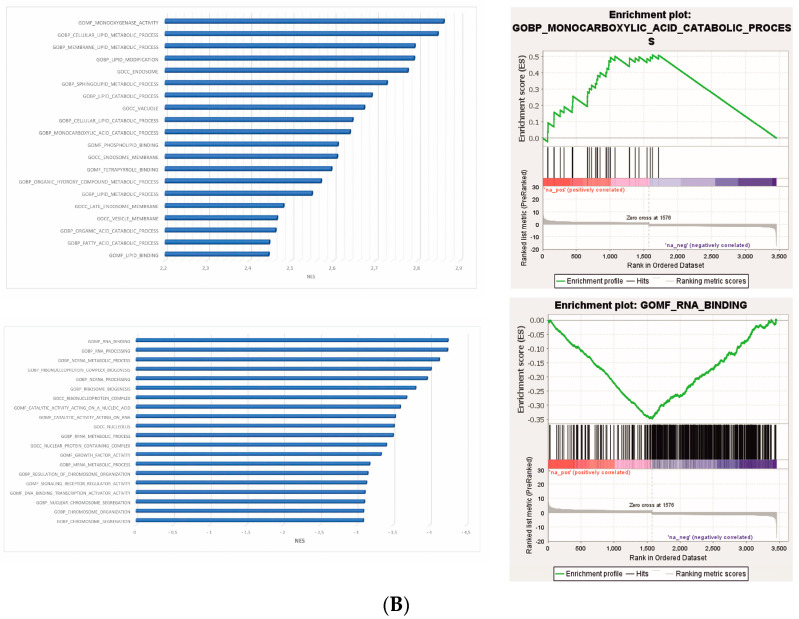
GSEA plots. (**A**) Bubble plot of the top 40 significant GO terms enriched in GSEA. In the plot, each GO term is defined in three distinct numerical parameters: the normalized enriched score (NES), nominal *p*-value and gene set size. In light green, the more significant nominal *p*-values are highlighted. (**B**) Histogram of GO terms with a positive or negative NES (top and bottom in the figure panel, respectively). Enrichment plot of the most significantly upregulated GO terms with positive or negative scores (top and bottom in the figure panel, respectively). The score at the peak of the plot is the enrichment score (ES) for the gene set.

Among the 40 significant Gene Ontology (GO) terms filtered by the normalized enrichment score (NES) and false discovery rate (FDR) (Figure 2A) (Table S4), the most significantly upregulated GO term in the phenotype na_neg was “molecular function RNA binding”. Conversely, in the phenotype na_pos, the most significantly upregulated Go term was “morphological functions monooxygenase activity” (Figure 2B) (Table S4).

**Figure 3:**
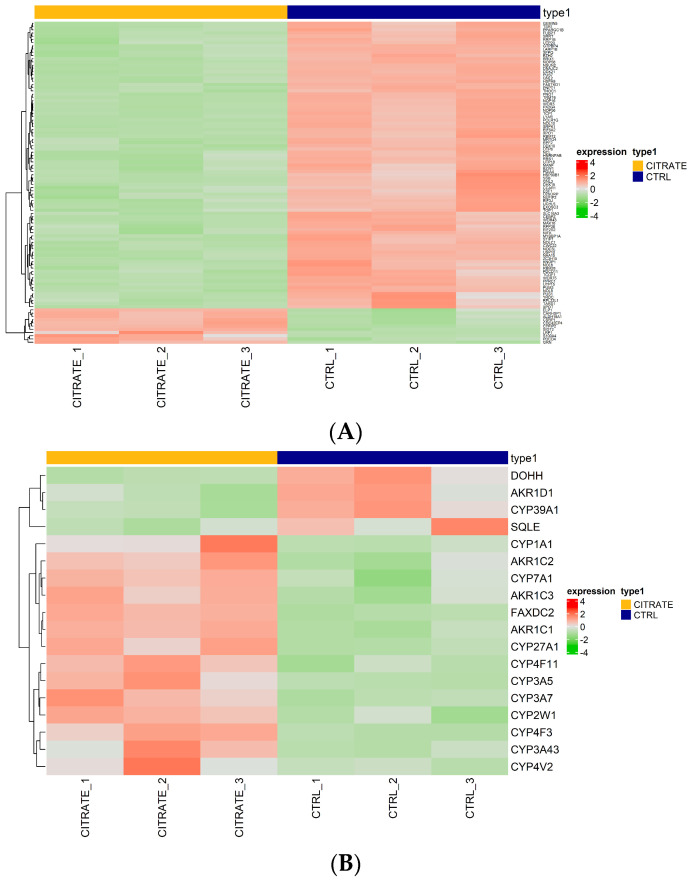
Expression heatmap. (**A**) Heatmap of differentially expressed genes ranked highest in the negative regulation of the GO term “molecular function RNA binding”. (**B**) Heatmap of differentially expressed genes ranked highest in the positive regulation of the molecular function GO term “morphological functions monooxygenase activity”. Red indicates that the expression level of the gene is relatively upregulated, and green indicates that the expression level of the gene is downregulated.

Regarding the genes involved in the “molecular function RNA binding”, some genes, such as *GEMIN5*, *TSR1* and *FUBP1*, were found to be downregulated compared to the controls (Figure 3A, Table S5). This suggests the potential modulation of RNA-related processes in the treated cells. The genes *GEMIN5*, *TSR1* and *FUBP1* are involved in snRNP biogenesis, ribosomal synthesis and post-transcriptional gene expression regulation, respectively [38,39,40]. Their downregulation may negatively affect RNA binding and regulation processes, potentially impacting protein synthesis and other RNA-dependent cellular mechanisms (Figure 3A).

Then, we focused our attention on genes involved in “morphological functions monooxygenase activity”, the most significantly upregulated GO term. Several genes were found to be upregulated in the treated cells compared to the controls (*CYP3A43*, *CYP3A5*, *AKR1C1*, *CYP1A1*, *FAXDC2*, *AKR1C2*, *CYP4F3*, *CYP7A1*, *CYP2W1*, *AKR1C3*, *CYP4V2*, *CYP27A1*, *CYP4F11*), among which 10 belonged to the cytochrome P450 family. Meanwhile, the genes *AKR1D1*, *SQLE*, *DOHH* and *CYP39A1* were found to be downregulated (Figure 3B, Table S6).

### 2.3. Validation of RNA-Seq Data by RT-qPCR Analysis

**Figure 4:**
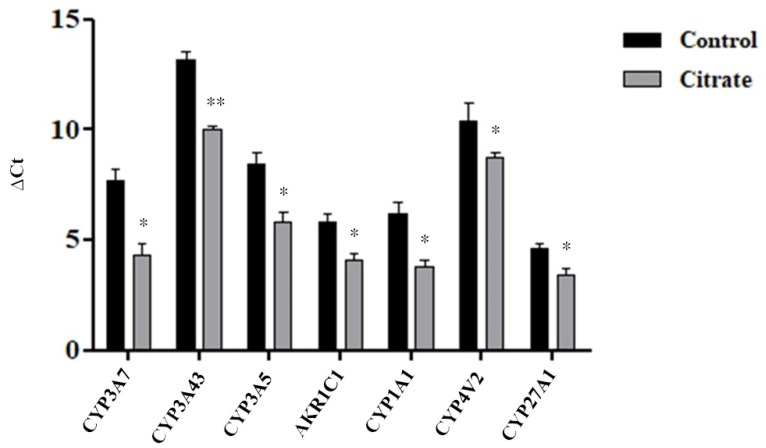
Expression levels of some genes involved in the “monooxygenase activity” pathway. The histograms illustrate the mean Ct value ± standard error (SE) of three independent experiments. The statistical analysis was performed using the GraphPad Prism 8.4.2 software (unpaired, two-tailed *t*-test, * *p*< 0.05, ** *p* < 0.01).

We evaluated, by the RT-qPCR assay, the expression levels of seven DEGs belonging to the “monooxygenase activity pathway”, for which the highest NES value was calculated. As shown in Figure 4 and in Table 1, the RT-qPCR data were generally consistent with the RNA-Seq results.

### 2.4. Effect of Extracellular Citrate on Migration and Invasion of HepG2 Cells

**Figure 5:**
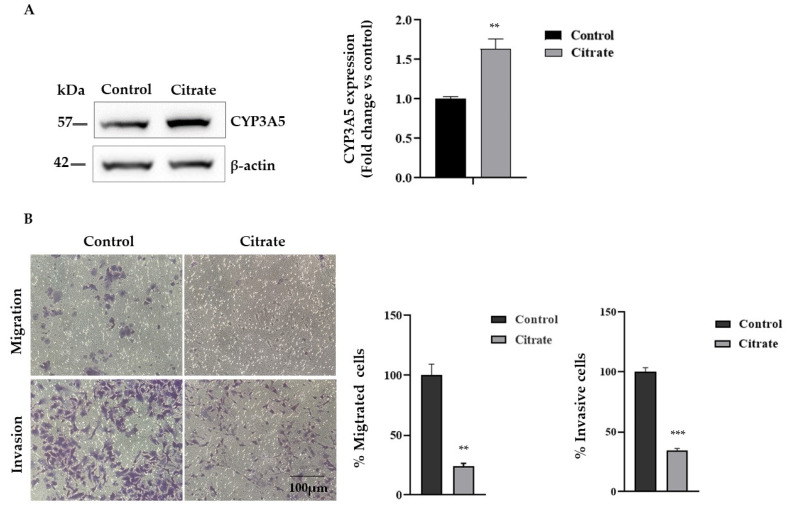
Migration and Matrigel invasion assay. (**A**) Representative Western blotting and densitometric analysis of CYP3A5 protein upon citrate treatment. β-actin was used as a loading control. Data, expressed as the mean ± SE of four independent experiments, are shown as a fold change compared to control cells. (**B**) Effect of citrate treatment on the invasive and migratory ability of HepG2 cells by Transwell assay (scale bar, 100 μm). Migrating and invaded cells are represented as purple cells dyed with crystal violet. Data are presented as the mean ± SE of three independent experiments. The statistical analysis was performed using the GraphPad Prism 8.4.2 software (unpaired, two-tailed *t*-test, ** *p* < 0.01, *** *p* < 0.001).

Since citrate treatment upregulates the expression of *CYP3A5*, which has an important role as a tumor suppressor in HCC, we evaluated the migration and invasion capacity of HepG2 cells treated with citrate. First, we analyzed the CYP3A5 protein expression, confirming a significant expression change (Figure 5A). Then, we examined the migration and invasion abilities of HepG2 cells exposed to 10 mM citrate for 24 h by the Transwell assay in vitro. As shown in Figure 5B, citrate treatment reduced migration and invasion compared with untreated cells by about 70% and 65%, respectively.

## 3. Discussion

Despite significant progress in cancer research and some improvements in therapeutic applications, metastatic cancer remains one of the deadliest diseases. In particular, HCC frequently spreads to the lungs, lymph nodes, adrenal gland and bones, including the skull, and the overall prognosis of patients with metastatic HCC is poor.

The transformation from a normal cell to a tumor cell occurs through mutations in specific genes, namely oncogenes and tumor suppressors, prompting the reprogramming of the cellular metabolism to meet the metabolic demands of the tumor cell. Therefore, any molecule capable of modifying the metabolic programming of cancer cells is to be considered a potential anticancer drug.

Metabolic reprogramming has been recognized as an emerging hallmark of cancer and has attracted much interest since it is intimately connected to epigenetic modifications, as it provides substrates or cofactors for different epigenetic enzymes, thus contributing to the development and progression of cancer [41,42]. Histone modifications, such as acetylation, methylation and phosphorylation, are crucial for molecular diversity, and their dysregulation is central to many diseases, including cancer [43]. Recent evidence suggests that histone acetylation is a common feature of hepatocellular carcinoma, playing pivotal roles in processes like proliferation, apoptosis, metastasis, metabolic reprogramming and drug sensitivity [44].

Although several studies have demonstrated that high concentrations of citrate (10–20 mM) inhibit cancer cell proliferation through several proposed mechanisms, the role of citrate in metastatic progression has not been well elucidated [20,26].

Gene expression analysis can monitor how genes respond to treatments, such as compounds or drugs. RNA-Seq utilizes the advantages of high-throughput sequencing by next-generation sequencing (NGS) and allows us to detect and quantify the RNA in a biological sample and therefore identify entire patterns of differentially expressed genes. In order to shed light on the effects on gene expression regulation caused by citrate supplementation, we treated HepG2 cells with 10 mM of citrate and verified, through gene transcription analysis, that a large number of genes (3551) were dysregulated, of which 851 were upregulated and 822 downregulated. In addition, cell motility assays showed that the citrate-treated cells migrated more slowly and were less invasive than the control cells. Given the high number of genes dysregulated by citrate treatment, it is difficult to specifically attribute the change in phenotype to just a few metabolic pathways. However, among the most upregulated gene families that could contribute to phenotypic characterization is the cytochrome P450 (CYPs) family, which includes enzymes involved in the bioactivation and detoxification of endogenous and exogenous compounds, cellular metabolism and homeostasis [45]. In particular, *CYP3A5*, *CYP3A7* and *CYP3A43,* which are members of the *CYP3A* subfamily, found in a gene cluster on chromosome 7, were overexpressed in HepG2 cells exposed to exogenous citrate. It has been shown previously that low expression levels of *CYP3A5* and *CYP3A43* are linked to a poor prognosis in HCC [46,47], and *CYP3A5* functions as a tumor suppressor gene in HCC by regulating the mTORC2/Akt signaling pathway [48]. Furthermore, the overexpression of *CYP27A1* and *CYP3A43* has been shown to reduce cell proliferation and migration in renal cell carcinoma and lung adenocarcinoma, respectively [49,50]. Taken together, these reports support the idea that it is the induction of CYP expression that leads to the decrease in cell migration and invasion. Therefore, the results presented in this study demonstrate that CYP expression may be induced by citrate treatment, which ultimately leads to reduced cell migration and invasion.

Numerous other studies have reported that several enzymes involved in lipogenesis are upregulated within HCC nodules, including fatty acid synthase (FASN) [39,51,52]. Transcriptomic analyses have revealed that several genes involved in cancer tumorigenesis and metastasis are downregulated in citrate-treated HepG2 cells, such as *FASN*, *Hexokinase 2* (*HK2*), *glucose-regulated protein-78* (*GRP78*, *HSPA5*), *forkhead box C1 protein* (*FOXC1*), *B-cell lymphoma/leukemia-2* (*Bcl-2*) and *myeloid cell leukemia 1* (*MCL-1*) [32,53,54,55,56,57] (Table S1). These observations suggest that citrate treatment causes a large variety of specific effects on different levels in metabolism, intracellular signaling pathways and gene expression regulation, mostly connected to metabolic reprogramming and/or apoptotic pathways. Therefore, at present, it appears difficult to deduce the main mechanism by which citrate supplementation leads to reduced cancer cell proliferation and metastatic progression. However, given the previously mentioned effects of the level of CYP expression in carcinomas [38,39,40,41,42], our findings in citrate-treated HepG2 cells strongly suggest that the induction of CYP expression plays a central role in cell migration, but further studies are warranted to deduce the main mechanism in which all observed effects may be connected. These data, on one hand, confirm the role of citrate in the regulation of different metabolic and epigenetic processes, and, on the other hand, demonstrate that cancer cells are particularly sensitive to changes in gene expression that can be induced by metabolites or xenobiotics

In conclusion, the findings of this study demonstrate that treatment of HepG2 cells with 10 mM citrate leads to extensive alterations in gene expression regulation, with the ultimate result of determining a less invasive phenotype of HepG2 cells. Therefore, citrate and CYPs may become important tools and targets in efficient gene reprogramming approaches for the development of future treatments aimed at limiting metastasis and fighting cancer.

## 4. Materials and Methods

### 4.1. Materials

Dulbecco’s modified eagle’s medium was purchased from Corning (Corning, New York, NY, USA). The trypsin–EDTA solution, Bradford reagent and sodium citrate were purchased from Sigma Aldrich–Merck (Saint Louis, MO, USA). Dulbecco’s phosphate buffered saline, L-glutamine, penicillin–streptomycin solution and fetal bovine serum were purchased from EuroClone (Milan, Italy). The primary antibody specific to the CYP3A5 (13737-1-AP) protein was purchased from Proteintech (Rosemont, IL, USA). Human hepatocellular carcinoma cell lines were obtained from the American Type Culture Collection (ATCC, Manassan, VA, USA).

### 4.2. Cell Culture and Treatments

Human hepatocellular carcinoma cell line HepG2 was cultured in Dulbecco’s modified eagle’s medium (DMEM), supplemented with 10% fetal bovine serum, 100 U/mL penicillin/streptomycin and 2 mM L-glutamine, at 37 °C with 5% CO_2_. All experiments were performed using cells with less than 10 passages, in the logarithmic growth phase. Sodium citrate was dissolved in distilled water to obtain stock solutions (1 M). HepG2 cells were seeded into 6-well plates at a density of 5 × 10^5^ cells per well and treated for 24 h at 37 °C with 10 mM sodium citrate. For the transcriptomic analysis, three biological replicates were prepared for each condition, i.e., citrate-treated and control cells.

### 4.3. RNA Extraction and Sequencing

Total RNA was extracted using the Quick-RNA MiniPrep kit (ZymoResearch, Irvine, CA, USA), according to the manufacturer’s instructions. The RNA concentration and purity were evaluated using a NanoDrop™ 2000/2000c (Thermo Fisher Scientific, Waltham, MA, USA), whereas the sample integrity was analyzed using the Tape Station 4200 (Agilent Technologies, Santa Clara, CA, USA) using an RNA Screen Tape Assay. Indexed libraries were prepared from 1 µg/ea purified RNA using the TruSeq Stranded mRNA (Illumina, San Diego, CA, USA) Library Prep Kit, according to the manufacturer’s instructions. After the enrichment of mRNA using oligo dT magnetic beads and fragmentation, cDNA synthesis was performed, followed by adapter ligation and PCR amplification. For library quantifications, the TapeStation 4200 (Agilent Technologies) was used. Indexed libraries were pooled in equimolar amounts, with a final concentration of 1.5 nM. To sequence the pooled samples, the Illumina NextSeq 550 DX System was used, in a 2 × 75 paired-end module. The raw sequence files generated (fastq files) underwent quality control analysis using FastQC [58]. The Cutadapt software (v.2.8) [59] was used to trim the short reads (≤25 bp) and adapter sequences. The next step was performed to map the fastq files on the reference genome, through the bioinformatics software STAR (version 2.7.3a) [60], with the standard parameters for paired reads. The reference track was the Human assembly obtained from GenCode (HG38—Release 37 (GRCh38.p13)) [61]. The quantification of the genes expressed for each sequenced sample was computed using the featureCounts algorithm (v 2.0) [62]. An ad hoc script in R was used to normalize the data and to find the genes differentially expressed in the samples using the Bioconductor DESeq2 package [63]. Genes showing a fold change ≥ 1.50 or ≤−1.50 (|FC| ≥ 1.50), along with adjusted *p*-values ≤ 0.05 (padj), were considered as differentially expressed. The ComplexHeatmap [64], ggplot2 [65] and EnhancedVolcano (https://doi.org/10.18129/B9.bioc.EnhancedVolcano, accessed on 17 May 2024) packages in R were used to create heatmaps and volcano plots of the differentially expressed genes, respectively. Functional analysis was performed on the statistically significant genes using the GSEA software (v.4.1.0) [65,66,67]. The RNA-seq experimental design is reported in Figure S1.

### 4.4. Reverse Transcription Quantitative PCR (RT qPCR)

The total RNA was transcribed to cDNA using oligo (dT) primers and the GeneAmp RNA PCR Core Kit (Applied Biosystems, Waltham, MA, USA), and the cDNA was amplified via real-time PCR using the Power-SYBR Green PCR Master Mix (Promega, Madison, WI, USA) on the 7500 Fast Real-Time PCR System (Applied Biosystems). Primers were designed with the Allele ID program to span exon–exon junctions, eliminating undesirable genomic DNA amplifications, and were purchased from Eurofins; they are listed in Table 2. Gene expression was analyzed according to 2^−ΔΔCt^ relative quantification method.

### 4.5. Western Blot Analysis

After citrate treatment, the HepG2 cells were lysed in ice-cold RIPA buffer (Cell Signaling Technology CST, Danvers, MA, USA) supplemented with a protease inhibitor cocktail and the protein concentration was detected using the Bradford assay, according to the manufacturer’s instructions. Samples were loaded to SDS-PAGE and transferred to nitrocellulose membranes. After incubation for 1 h in blocking buffer (5% *w*/*v* non-fat dry milk in TRIS-buffered saline, pH 7.4, supplemented with Tween 20 0.1% (*v*/*v*)), the membrane was incubated with primary antibody anti-CYP3A5 diluted 1:600 in blocking buffer at 4 °C overnight and subsequently in an appropriate HRP-conjugated secondary antibody. Chemiluminescence was detected using the ECL Star Enhanced Chemiluminescent Substrate and LiteAblot TURBO Extra Sensitive Chemiluminescent Substrate (EuroClone, Milan, Italy), through the Chemidoc XRS detection system (BioRad, Hercules, CA, USA), with the ImageLab 5.1 software (Bio-Rad). Densitometric data were collected by the Image J software (https://imagej.net/ij/ accessed on 1 January 2024) (National Institutes of Health) for further statistical analysis.

### 4.6. Transwell Assay for Migratory and Invasive Cell Properties

For the migration assay, HepG2 cells were cultured in serum-free medium for 24 h before being added to the upper chamber of a non-coated 24-well Transwell plate (pore size: 8 µm, Sterlitech Corporation, Auburn, MI, USA, Washington, DC, USA). The Transwells were coated with Matrigel 100 μg/mL (Corning Life Sciences, Shanghai, China) for the cell invasion experiment. DMEM supplemented with 10% FBS was added to the lower chamber as a chemoattractant. After 24 h of incubation at 37 °C with 10 mM sodium citrate and the control (distilled water), the cells were fixed with 4% paraformaldehyde for 10 min at room temperature and stained with 0.1% crystal violet for 10 min. Non-invading cells were removed from the upper surface with cotton swabs. A light microscope was used to photograph the cells. The experiments were carried out in triplicate and the percentage of migratory and invasive cells was expressed as the mean ± SE.

### 4.7. Statistical Analysis

Results are reported as the mean values of least three independent experiments ± standard error (SE). Data were analyzed using GraphPad Prism (version 8.4.2, GraphPad Software, San Diego, CA, USA) using an unpaired two-tailed *t*-test, considering *p*-values < 0.05 as statistically significant.

## Figures and Tables

**Table 1:** RNA-Seq versus RT-qPCR gene expression correspondence.

Gene	Fold Change RNA-Seq	*p*-Value RNA-Seq	Fold Change RT-qPCR	*p*-Value RT-qPCR
*CYP3A7*	7.61	4.45 × 10^−8^	10.2	0.0104
*CYP3A43*	5.93	1.91 × 10^−4^	8.76	0.0019
*CYP3A5*	5.82	4.48 × 10^−16^	6.03	0.0258
*AKR1C1*	3.53	5.14 × 10^−7^	3.31	0.0253
*CYP1A1*	2.68	8.11 × 10^−4^	5.22	0.015
*CYP4V2*	1.67	4.15 × 10^−3^	3.28	0.019
*CYP27A1*	1.56	9.67 × 10^−8^	2.28	0.0373

Fold change = ratio of normalized expression absolute value in sample over control.

**Table 2:** Primer sequences used for RT-PCR analysis.

Gene	Accession Number	Forward Primer (5’to 3’)	Reverse Primer (5’to 3’)
*β-actin*	NM_001101.3	CCTGGCACCCAGCACAAT	GCCGATCCACACGGAGTACT
*CYP3A7*	NM_000765.5	ATCATTGCTGTCTCCAACATTCAC	GCTTGCCTGTCTCTGCTTCC
*CYP3A43*	NM_022820.5	ATGGTTCCAATCTATGCTCTTCAC	ATGCTGTCCTTGTTCTTCTTACTG
*CYP3A5*	NM_000777.5	TCATTGCTGTCTCCAACCTTCAC	GCTTGCCTTTCTCTGCTTCCC
*AKR1C1*	NM_001353.6	TGTAAAGCCAGGTGAGGAAGTG	CTGCGGTTGAAGTTGGACAC
*CYP1A1*	NM_000499.5	ATCCCTATTCTTCGCTACCTACCC	GTGCTCCTTGACCATCTTCTGC
*CYP4V2*	NM_207352.4	AGTCTGACCGTCCCGCTAC	GTAACCTGCCACTTCACAATCTTC
*CYP27A1*	NM_000784.4	GCCAGTGCCCCGCTCTTG	TGGTGTCCTTCCGTGGTGAAC

*β-actin*: beta actin; *CYP3A7*: cytochrome P450 3A7; *CYP3A43*: cytochrome P450 3A43; *CYP3A5*: cytochrome P450 3A5; *AKR1C1*: aldo-keto reductase family 1-member C1; *CYP1A1*: cytochrome P450 1A1; *CYP4V2*: cytochrome P450 4V2; *CYP27A1*: cytochrome P450 27A1.

## Data Availability

The data presented in this study are available from the corresponding authors upon request.

## Supplementary Materials

The following supporting information can be downloaded at: https://www.mdpi.com/article/10.3390/ijms25126509/s1.
